# Occurrence of Antimicrobial-Resistant *Enterococcus* spp. in Healthy Chickens Never Exposed to Antimicrobial Agents in Central Italy

**DOI:** 10.3390/antibiotics13050417

**Published:** 2024-05-01

**Authors:** Giulia Cagnoli, Alessia Di Paolo, Fabrizio Bertelloni, Sonia Salvucci, Arianna Buccioni, Margherita Marzoni Fecia di Cossato, Valentina Virginia Ebani

**Affiliations:** 1 Department of Veterinary Science, University of Pisa, Viale Delle Piagge 2, 56124 Pisa, Italy; giulia.cagnoli@phd.unipi.it (G.C.); alessia.dipaolo@studenti.unipi.it (A.D.P.); fabrizio.bertelloni@unipi.it (F.B.); sonia.salvucci@unipi.it (S.S.); margherita.marzoni@unipi.it (M.M.F.d.C.); 2 Interdepartmental Research Center “Nutraceuticals and Food for Health”, University of Pisa, Via Del Borghetto 80, 56124 Pisa, Italy; 3 Department of Agriculture, Food, Environment and Forestry, University of Florence, Piazzale Delle Cascine 18, 50144 Florence, Italy; arianna.buccioni@unifi.it; 4 Centre for Climate Change Impact, University of Pisa, Via Del Borghetto 80, 56124 Pisa, Italy

**Keywords:** *Enterococcus*, poultry, antibiotic resistance, multidrug resistance, resistance genes

## Abstract

Enterococci are part of the natural flora of the gastrointestinal tract of mammals, including humans, birds and invertebrates. They can cause infection, mainly among hospitalized patients, as well as acquire and transfer antimicrobial resistance genes. The present study allowed the isolation of 98 *Enterococcus* (73.47% *E. faecium*, 23.47% *E. faecalis*, 3.06% *E. avium*) strains from 120-day-old healthy chickens that had never been treated with antimicrobials. Their antimicrobial resistance was evaluated by the agar disk diffusion method; high-level aminoglycoside (streptomycin and gentamicin) and vancomycin resistance were established using the microbroth dilution method. The highest percentages of resistant isolates were detected with quinupristin–dalfopristin (88.78%), rifampicin (64.29%), tetracyclines (45.92%), and enrofloxacin (41.84%). High percentages of susceptible strains were found with teicoplanin (100%), amoxicillin–clavulanic acid (97.96%), nitrofurantoin (94.90%), ampicillin (92.86%), chloramphenicol (90.82%), and linezolid (88.78%). About 60% of the strains were classified as MDR (multidrug-resistant). Moreover, PCR was carried out to investigate genes encoding for tetracyclines resistance determinants: *tet*(M), *tet*(L), *tet*(O), *tet*(K), and *Int-Tn*. Genes were detected in 68 (69.38%) strains: 36 were shown to be resistant with the agar disk diffusion method, while 28 were intermediate, and 2 were susceptible. The present study showed that chickens never treated with antimicrobials potentially harbor enterococci having phenotypic and genotypic characters of antimicrobial resistance.

## 1. Introduction

*Enterococcus* bacteria are Gram-positive commensals of the microbiota of numerous animal species, including humans; they are known to cause various infections in animals, including mastitis in cattle, bacteraemia in dogs and pigs [1,2], and septicaemia, endocarditis, amyloid arthropathy, and spondylitis in poultry [3,4].

Widely recognized as important agents of nosocomial human infections, they carry intrinsic antibiotic resistance for some antibacterial molecules; moreover, they have a strong ability to acquire, express and transfer genes coding for antimicrobial resistance [5,6,7]. In particular, in the gastrointestinal habitat, enterococci are in a suitable position to acquire resistance genes from other commensals, which may further transfer to other more pathogenic bacteria [8].

Antimicrobial resistance is a significant global public health matter that poses a threat to both human and animal populations. The emergence and spread of antimicrobial resistance among *Enterococcus* spp. is a growing concern due to its implications for animal and public health under the One Health framework. The use of antibiotics in food-producing animals, such as poultry, raises critical concerns regarding food safety, animal health, and the potential transmission of genes coding for antimicrobial resistance to humans [9]. 

There is a substantial scientific literature regarding the antimicrobial resistance of the genus *Enterococcus* in many animal species including wild birds [10,11] and poultry such as broilers, hens, turkeys, and ducklings from different global regions [9,12,13,14,15,16,17]. The different surveys conducted to evaluate the antimicrobial-resistant enterococci prevalence in poultry detected different rates, which are often difficult to compare with each other, in relation to several factors, such as animal population (age, health status, production), breeding condition, tested antimicrobials, and geographic area. However, based on the collected data, antimicrobial-resistant enterococci are currently recognized as an emerging problem in the poultry industry, and their role is yet to be fully understood [17]. 

Previous studies on poultry were usually focused on enterococcal infections in diseased birds [3,4] or in healthy animals bred in farms for commercial purposes [12,13,14,15,16].

The present study aimed to investigate the phenotypic and genotypic antimicrobial resistance characters of *Enterococcus* strains isolated from cloacal swabs of healthy young chickens that had never been treated with antibiotics or coccidiostats. The animals involved in the study did not belong to farms; they were bred in small structures of the University of Pisa and University of Florence for breeding, and they were not commercial in order to preserve the biodiversity of some Italian breeds.

## 2. Results

### 2.1. Enterococcus spp. Isolation and Typing 

A total of 98 *Enterococcus* spp. strains were isolated and typed at species level: 72 (73.47%; 95% CI: 64.63–82.21%) *E. faecium*, 23 (23.47%; 95% CI: 15.08–31.86%) *E. faecalis*, and 3 (3.06%; 95% CI: 0.00–6.47%) *E. avium.*

### 2.2. Antimicrobial Susceptibility Tests

All the examined isolates (98/98, 100%) were shown to be susceptible to teicoplanin with the agar disk diffusion test. High percentages of susceptible strains were found with amoxicillin–clavulanic acid (96/98, 97.96%), nitrofurantoin (93/98, 94.90%), ampicillin (91/98, 92.86%), chloramphenicol (89/98, 90.82%), and linezolid (87/98, 88.78%).

The highest percentages of resistant isolates were detected with quinupristin–dalfopristin (87/98, 88.78%) and rifampicin (63/98, 64.29%); high percentages of strains were resistant to tetracyclines (45/98, 45.92%) and enrofloxacin (41/98, 41.84%). Moreover, 42.86% (42/98) and 41.84% (41/98) of isolates were intermediate to enrofloxacin and ciprofloxacin, respectively. The results obtained by the agar disk diffusion tests are summarized in Table 1, Table 2, Table 3 and Table 4.

In the HLAR assay, among the 98 tested strains, only one (1.02%; 95% CI: 0.00–3.01%) *E. faecium* isolate was found to be resistant to both streptomycin (HLSR) and gentamicin (HLGR). Four (4.08%; 95% CI: 0.16–8.00%) and 2 (2.04%; 95% CI: 0.00–4.84%) *E. faecalis* isolates demonstrated HLSR and HLGR, respectively. 

No specific resistance levels to vancomycin were observed among the strains shown to be resistant or intermediate in the disk diffusion test when evaluated for minimum inhibitory concentration. 

On the basis of all obtained results, 58/98 (59.18%; 95% CI: 49.45–68.91%) isolates were classified as multidrug resistant (MDR) (non-susceptible to ≥1 agent in ≥3 antimicrobial classes): 45/72 (62.50%; 95% CI: 52.91–72.09%) *E. faecium*, 12/23 (52.17%; 95% CI: 31.75–72.59%) *E. faecalis* and 1/3 (33.33%; 95% CI: 0.00–86.67%) *E. avium*; no statistical differences emerged (*p* > 0.05). The remaining 40 (40/98, 40.81%; 95% CI: 31.08–50.54%) strains were identified as belonging to the no-resistance class.

### 2.3. Genotypic Resistance 

As regards the analysis of tetracycline resistance genes, 68 (69.38%; 95% CI: 60.25–78.51%) strains carrying resistance genes were identified in the total of 98 strains analyzed. In detail, 64 (65.30%; 95% CI: 55.88–74.72%) strains had *tet*(M), 10 (10.20%; 95% CI: 4.21–16.19%) *tet*(L), and 2 (2.04%; 95% CI: 0.00–4.84%) *tet*(O), whereas no strains carried the *tet*(K) gene. Thirty (30.61%; 95% CI: 21.49–39.73%) strains were negative for all investigated genes. Twelve (12.24%; 95% CI: 5.75–18.73%) strains positive to the *Int-Tn* gene were identified as always associated to *tet*(M); eight (8.16%) strains tested positive for the *tet*(M) and *tet*(L) genes. The results of the molecular analyses are reported in Table 5.

Regarding the comparison between phenotypic and genotypic resistance to tetracycline, out of the 45 strains that were found to be phenotypically resistant, 36 (80%; 95% CI: 68.31–91.69%) tested positive for at least one of the target genes. Of these, 34 (94.44%; 95% CI: 66.93–93.07%) samples tested positive for the *tet*(M) gene, 5 (13.88%; 95% CI: 2.59–25.17%) for the *tet*(L) gene, 2 (5.55%; 95% CI: 0.00–13.03%) for the *tet*(O) gene and 9 (25%; 95% CI: 10.85–39.15%) for the *Int-Tn* gene. The *tet*(M) gene was also detected in 28 (75.67%; 95% CI: 61.84–89.50%) of the 37 intermediate strains, and 3 of these were associated with the *Int-Tn* gene. One (2.7%; 95% CI: 0.00–7.92%) intermediate strain had only *tet*(L) gene, while 2 (5.4%; 95% CI: 0.00–12.68%) intermediate strains had both *tet*(M) and *tet*(L). Of the 16 phenotypically susceptible strains, 2 were found to be positive for both the *tet*(M) and *tet*(L) genes.

### 2.4. Resistance Patterns

The 72 *E. faecium* strains showed 28 different phenotypic resistance patterns, which were shown to be resistant from 1 to 9 different antimicrobials (Table 6). Although no resistance cluster prevailed, 54/72 (75.00%; 95% CI: 65.00–85.00%) *E. faecium* strains showed the resistance profile for rifampicin/quinupristin–dalfopristin. Whereas 33/72 (45.83%; 95% CI: 34.32–57.34%) displayed the resistance profile for quinupristin–dalfopristin/tetracycline, and 28/72 (38.88%; 95% CI: 27.62–50.14%) showed the resistance profile for rifampicin/quinupristin–dalfopristin/tetracycline.

The 23 *E. faecalis* strains showed 14 different phenotypic resistance patterns, which were resistant from 1 to 7 different antimicrobials; four strains were susceptible to all tested antimicrobials (Table 7). Although no resistance cluster prevailed, 10/23 (43.47%; 95% CI: 23.21–63.73%) *E. faecalis* strains showed the resistance profile erythromycin/quinupristin–dalfopristin, whereas 9/23 (39.13%; 95% CI: 19.18–59.08%) displayed the resistance profile rifampicin/quinupristin–dalfopristin.

The three *E. avium* strains showed 2 different phenotypic resistance patterns, which were shown to be resistant from 1 to 6 different antimicrobials (Table 8).

The resistance profiles rifampicin/quinupristin–dalfopristin and rifampicin/quinupristin–dalfopristin/tetracycline were more frequently encountered in *E. faecium* strains than in *E. faecalis* strains (*p* < 0.5). Meanwhile, the resistance profile erythromycin/quinupristin–dalfopristin was more often associated to *E. faecalis* than *E. faecium* (*p* < 0.5). The resistance profile quinupristin–dalfopristin/tetracycline was equally distributed among the 2 bacterial species (*p* > 0.5).

## 3. Discussion

The animals involved in the study were not from a farm; they belonged to a small group of chickens bred in the university facilities for a project focused on the poultry biodiversity preservation. Our study had not epidemiological purpose but aimed to investigate enterococci present in the intestine of these animals, which were healthy and have never been treated with antibiotics or coccidiostats. 

*Enterococcus faecium* and *E. faecalis* were the species most frequently isolated with prevalences of 73.47% and 23.47%, respectively, which is in agreement with a previous study on laying hens from the same geographic area [18]. Other authors identified *E. faecium* as the most encountered species in poultry [19,20]; conversely, *E. faecalis* was the most frequently isolated enterococcal species in other investigations [9,21,22,23,24]. The different findings could be related to the tested samples, because some studies focused on specimens collected from chicks only a few days old, in which *E. faecalis* is the primary gut host [23,25]. Furthermore, some studies focused on chickens that exhibited clinical symptoms [22]; in fact, *E. faecalis* is considered a species more frequently associated to illnesses in poultry compared to *E. faecium* [16]. *Enterococcus avium* was scarcely present (3.06%) in the intestinal tract of the chickens tested in our study, which aligns with previous surveys in poultry [9,23]. 

The results of the agar disk diffusion test highlighted the frequent antimicrobial resistance among enterococci. Only four strains, all *E. faecalis*, isolated in this study were not resistant to any substance. 

Overall, the greatest percentage of resistant isolates was observed with the streptogramins quinupristin/dalfopristin (88.77%) and rifampicin (64.29%). A relevant percentage of resistant isolates was also found with tetracycline (45.92%). However, the percentages of non-susceptible isolates increase if also strains with intermediate sensitivity are included. Therefore, more than 80% of the analyzed strains were not susceptible to enrofloxacin and tetracycline, and more than half were not susceptible to ciprofloxacin. On the other hand, 100% of the tested strains were susceptible to glycopeptides; in detail, all isolates were susceptible to teicoplanin with the Kirby–Bauer test, and 11 strains were shown to be resistant to vancomycin with this assay, which successively showed susceptibility with the MIC determination. Furthermore, high percentages of susceptible isolates were detected with penicillins (97.96% to amoxicillin–clavulanic acid and 92.86% to ampicillin) as well as with nitrofurantoin (94.90%). 

HLAR was tested on all isolates; only one *E. faecium* strain showed high-level resistance to both streptomycin and gentamicin, whereas four and two *E. faecalis* isolates were resistant to streptomycin and gentamycin, respectively. In the absence of HLAR, enterococci with lower resistance to cell wall active agents, such as penicillin or ampicillin, may be susceptible to the synergistic killing of an aminoglycoside–penicillin combination therapy [5]. 

On the basis of the results obtained by Kirby–Bauer, MIC, and HLAR assays, 59.18% of the analyzed strains were classified as MDR; this finding is quite in accordance with the 53% of MDR strains detected by Bertelloni et al. [18] but in contrast with the 33.3% found by Alzharani et al. [9] in investigations on enterococci from poultry. A correlation between the production of biofilm, a relevant pathogenicity character, and antibiotic resistance in enterococci, mainly in MDR strains, has been supposed [9,26]. 

The high percentage (88.78%) of enterococcal strains resistant to quinupristin/dalfopristin is in accordance with previous investigations in which different enterococcal species isolated from poultry frequently were shown to be resistant to streptogramins [18,27,28]. Quinupristin–dalfopristin is a water-soluble mixture of streptogramin A and B moieties. These two structurally unrelated molecules bind to bacterial ribosomes, acting synergically to inhibit protein synthesis at the elongation step. Quinupristin–dalfopristin is largely used in human clinical practice to treat infections due to multi-resistant organisms such as methicillin-resistant Staphylococcus aureus (MRSA) and vancomycin-resistant *E. faecium* [29]. Even though *E. faecalis* is considered intrinsically resistant to quinupristin–dalfopristin [29], in our study, *E. faecium* was statistically more frequently resistant (95.83%) to this antimicrobial compared to *E. faecalis* (78.26%). 

The overall resistance to rifampicin found in our survey was 64.29%, with a significant impact on *E. faecium*, which showed 79.17% resistance, in contrast to previous studies that found higher sensitivity rates [18,21]. Combinations of rifampicin with either ciprofloxacin, linezolid, daptomycin or tigecycline were shown to be effective against *E. faecalis* infection—mainly against the biofilm formation [30]. The rapid development of resistance against rifampicin is one of the major concerns in the treatment of enterococcal infection. Some authors hypothesized that *E. faecalis* rifampicin resistance could be limited in vitro by combining rifampicin with a fluoroquinolone or linezolid [30]. However, also, enterococcal resistance to linezolid is an increasing concern. Our study detected a small number (5.10%) of *Enterococcus* strains were shown to be linezolid-resistant, but this finding showed that resistance to this antimicrobial is possible, as also demonstrated in other surveys [31]. Currently, this is a relevant threat in human medicine because linezolid is usually used for the treatment of infections with clinically relevant Gram-positive bacteria harboring resistances, such as MRSA and vancomycin-resistant enterococci [32,33].

High non-susceptibility rates to fluoroquinolones were detected: 60.20% to ciprofloxacin and 84.69% to enrofloxacin. In the last few years, increasing numbers of fluoroquinolone-resistant enterococcal strains, of animal and human origin, have been observed. Previous studies in poultry detected high percentages of *Enterococcus* spp. resistant to this antimicrobial class [18,23,28]. *Enterococcus faecium* is considered naturally ciprofloxacin-resistant [28], but in our survey, only 16.67% of the isolates belonging to this species were shown to be resistant to ciprofloxacin, and 50% were classified as intermediate to the same antimicrobial. Fluoroquinolones are often used for the treatment of avian colibacillosis in poultry production, creating relevant concern about the potential spread of fluoroquinolone-resistant bacteria. For this reason, the European Medicines Agency’s Committee for Veterinary Medicinal Products suggested to revise the currently approved dose of enrofloxacin for poultry. The monitoring of enterococcal strains resistant to fluoroquinolones is very important, because these antibiotics, classified by the World Health Organization (WHO) as “critically important in human and veterinary medicine,” are pivotal for treating infections by pathogens, such as *Campylobacter, Salmonella* and *Escherichia coli* [34].

Resistance to chloramphenicol was found in 5.10% of the investigated isolates; in particular, 2.78% of *E. faecium* and 13.04% of *E. faecalis* strains were resistant, which was in agreement with the findings of a previous study in broiler breeders [35]. In fact, although chloramphenicol is currently banned from use in food-producing animals in many countries and regions, including the European Union, bacteria resistant to this antimicrobial are frequent [36]. Resistance to chloramphenicol is mainly caused by the production of inactivating chloramphenicol acetyltransferase, the genes of which are widely disseminated on plasmids and capable of contributing to multidrug resistance by conjugative transfer [36]. Therefore, the occurrence of chloramphenicol-resistant enterococcal strains is of particular interest, and its relation to MDR has been often studied [36,37,38]. 

All the investigated strains were susceptible to teicoplanin and vancomycin. Previous studies found low rates of enterococcal isolates from poultry resistant to glycopeptides [9,18,23]. Resistance to this antimicrobial class is constantly monitored because these molecules are pivotal in the treatment of human *Enterococcus* infections. Significant intercountry differences in the frequency of vancomycin-resistant Enterococcus strains have been documented: resistance rates of *E. faecium* are >70% in the USA [39], 16.8% in Europe [40], 34% in South Korea [41], and 1.4% in Japan [42]. Teicoplanin is also widely used, because it possesses more potent in vitro activity against enterococci than vancomycin, along with a longer half-life, which enables a once-daily dose [43]. 

The overall resistance rate to erythromycin was 15.31%; when considering only *E. faecalis* strains, the rate increased to 43.48%. Although enterococci are intrinsically susceptible to erythromycin, due to domain II and domain V of 23S rRNA, resistance to this antibiotic has been continuously reported due to acquired erythromycin-resistance genes, such as the ermB gene [44]. Macrolides, classified as critically important (the highest priority) for human medicine by the WHO, are largely employed in poultry breeders [9]; therefore, the monitoring of resistance to this antimicrobial class in enterococci of avian origin is necessary. 

In the present study, penicillins were shown to be active against the isolated enterococci: only 7.14% of the strains were resistant to ampicillin, whereas almost all strains were susceptible to amoxicillin–clavulanic acid. Our findings contrast with the fact that enterococci have been described as intrinsically resistant to β-lactam antibiotics, such as penicillins, carbapenems, and cephalosporins [45]. In fact, enterococci express low-affinity penicillin-binding proteins (PBPs) that are responsible for their weak binding to β-lactam antibiotics [46]. However, an increased production of PBPs has been associated with acquired resistance to penicillin or ampicillin among clinical *E. faecium* isolates [46]. *Enterococcus faecium* isolated from healthy poultry in Portugal have revealed a 30% rate of resistance to ampicillin [47], and a gene involved in this resistance has been identified in enterococci isolated from healthy broilers [9,24,48]. 

Tetracycline resistance is commonly detected in enterococci found in chicken samples [16,18,23,28,35,49]. Our investigation found 45.92% resistant strains and a very relevant percentage, 83.67%, of not-susceptible strains. Resistance to tetracycline is an important threat because these antimicrobials are largely employed in the treatment of animal and human infections. Moreover, the finding of about 30% of isolates resistant to tigecycline, a last-resort antimicrobial, is relevant, because it shows a gradually rising trend of resistance, as also observed by other studies [50].

The molecular analyses identified the presence of genes involved in the tetracycline resistance in 68 (69.38%) tested isolates. Most of the strains had *tet*(M), which was always in association with the *Int-Tn* gene. Additionally, 10 (10.20%) strains carried *tet*(L), and 2 (2.04%) carried *tet*(O). No strains had the *tet*(K) gene. 

All investigated genes are essential for resistance to tetracyclines. In particular, *tet*(M) and *tet*(O) genes encode for cytoplasmic proteins that protect ribosomes from the antibiotic, while *tet*(L) and *tet*(K) genes encode for membrane proteins that excrete the antibiotic [35,51]. The transposon integrase *Int-Tn* gene is also involved in tetracycline resistance diffusion: it encodes for the integrase enzyme associated with the insertion process of mobile genetic material [52]. 

The detected higher prevalences of *tet*(M) and *tet*(L) genes are not surprising, as these genes are the most involved in tetracycline resistance as previously observed in enterococci isolated from humans, animals, food, and the environment [9]. 

In detail, 80% (36/45) of the isolates that were shown to be tetracycline-resistant with Kirby–Bauer had at least one resistance gene, which was in accordance with the overall results obtained in previous studies [9,35]. 

Furthermore, the investigated genes were also found in strains that were classified as susceptible or intermediate based on phenotypic tests. In particular, *tet*(M) was observed in both sensitive and intermediate strains, while *tet*(L) and *Int-Tn* genes were detected in the intermediate strain. Other genes could be involved in tetracycline resistance; however, our finding, in agreement with previous studies, confirms that in some cases, enterococci can carry genes responsible for antimicrobial resistance without showing phenotypic resistance [53].

The different results obtaining by antimicrobial resistance analyses allowed us to identify several resistance patterns among the isolates, mainly the MDR strains. Different patterns were also detected within a given species, suggesting that more strains belonging to the same enterococcal species circulated among the investigated animals at the sampling time.

Our study has some limitations. First, even though the strength of the study comes from the analyses on a group of animals bred not for commercial purpose and never treated with antimicrobials, the number of the animals involved in the investigation is not high. In addition, the birds were not from farms; therefore, they could not reflect the situation in which farm chickens live. A second concern the samples we could collect did not allow clarifying some aspects, such as the moment at which the animals acquired the *Enterococcus* strains. However, it has been argued that part of the microbial colonizers harbored in early embryos were inherited from maternal hens, and the gut microbial abundance and diversity were influenced by environmental factors and host genetic variation during development [54]. Finally, further molecular analyses to detect other genes involved in the resistance mechanisms to tetracycline and other antimicrobials are necessary to better assess the role of enterococci as source of this genetic material for other bacterial strains. In this study, we focused on horizontally transferable resistance genes, in particular those for tetracycline resistance considering that this resistance is abundantly diffuse due to a large use of tetracyclines, especially in the past. 

## 4. Materials and Methods

### 4.1. Ethical Statement

Animal handling was carried out in accordance with Italian Government guidelines (D.lgs 26/2014). This study was approved by the Ethics Committee of Pisa University (Ref.: OPBA_33/2021), under article.2, paragraph.1, point b, of the Italian legislative decree n. 26/2014. No experimental procedures on growing chicken were performed. The study was exempt from ethical approval. 

### 4.2. Sampling 

Between June and July 2022, 124 cloacal swab samples were collected from 120-day-old chickens reared in the facilities of the Department of Veterinary Sciences, University of Pisa and in the experimental farm belonging to the University of Florence. The animals involved in the study and reared for breeding purpose belonged to four native Italian breeds (Livorno, Bianca di Saluzzo, Mugellese, and Valdarnese breed), representing slow-growing egg-type or double attitude chicken breeds, and were obtained from the Avian conservation centers of Pisa and Florence Universities. Chicks were reared in indoor wired cages up to thirty-five days of age; thereafter, they were moved to roofed outdoor pens on hay litter floor and under natural weather conditions (photoperiod from April to July, 13–15L:11–9D). Each outdoor pen contained about ten animals of the same breed; the pens were adjacent and only separated by a fence; all pens were roofed to limit the possible contamination with droppings of wild birds. All animals involved in the avian conservation project were enrolled for the study on enterococci. Birds were never treated with antibiotics, coccidiostats or other pharmacological substances. Chicks had free access to water and received the same standard feeding plan with inorganic unmedicated starter and grower diets. 

During the same sampling day, the swabs, kept at 4 °C, were transferred to the Avian Pathology Laboratories of the Department of Veterinary Sciences, University of Pisa where they were submitted to bacteriological analyses.

### 4.3. Enterococcus spp. Isolation 

Each swab was cultured in Buffered Peptone Water (BPW) (Oxoid Ltd., Basingstoke, UK) at 37 °C for 24 h. The culture was then transferred onto Kanamycin Aesculin Azide Agar (KAAA) plates and aerobically incubated at 42 °C for 24 h. A single isolated colony from each sample was subcultured on Tryptone Soy Agar (TSA) (Oxoid Ltd.) to obtain pure cultures and further processed. The isolates were subjected to species identification using the API 20Strep^®^ (Biomerieux, Marcy l’Etoile, France) in strict accordance with the manufacturer’s instructions. Isolated strains were stored in Brain–Heart Infusion (BHI) broth (Oxoid Ltd.), with the addition of 30% glycerol as a cryoprotectant, at −80 °C. 

### 4.4. Antimicrobial Susceptibility Tests 

The disk diffusion method was employed to test the resistance of 98 selected isolates [55]. The test was executed on Muller–Hilton agar (Oxoid Ltd.) plates. The isolates were tested with the following 14 antimicrobial disks (Oxoid Ltd.), belonging to 10 antibiotic classes: amoxicillin–clavulanic acid (20/10 µg), ampicillin (10 µg), chloramphenicol (30 µg), ciprofloxacin (5 µg), enrofloxacin (5 µg), erythromycin (15 µg), linezolid (30 µg), nitrofurantoin (300 µg), quinupristin–dalfopristin (15 µg), rifampicin (5 µg), teicoplanin (30 µg), tetracycline (30 µg), tigecycline (15 µg), and vancomycin (30 µg). The obtained inhibition zones were interpretated according to CLSI [56] and EUCAST [57] criteria. *Escherichia coli* ATCC 25922 and *Staphylococcus aureus* ATCC 25923 were used as quality controls in the disk diffusion test.

Isolates were tested for high-level aminoglycoside resistance (HLAR); the broth microdilution method suggested for this specific purpose by CLSI was adopted, and concentrations of 1000 µg/mL and 500 µg/mL were used as cut-off values for streptomycin and gentamicin, respectively [56]. 

The minimum inhibitory concentration (MIC) against vancomycin was determined for strains that were shown to be resistant or intermediate in the disk diffusion test. Vancomycin was diluted from 256 to 0.5 μg/mL; the breakpoint value was ≥32 µg/mL [58]. 

Based on the phenotypic resistance results, the investigated strains were classified as multidrug-resistant (MDR), extensively drug-resistant (XDR), or pan drug-resistant (PDR), as previously proposed by Magiorakos et al. [59]. 

### 4.5. Genotypic Resistance 

DNA extraction was carried out from each enterococcal isolate using the commercial kit DNA Plus Kits (Zymo Research, Irvine, CA, USA) and following the manufacturer’s guidelines. DNA samples were submitted to different polymerase chain reaction (PCR) assays to verify the presence of the following genes encoding for resistance to tetracyclines: *tet*(M), *tet*(L), *tet*(O), *tet*(K), and the presence of the integrase gene (*Int-Tn*) located on the Tn916–1545 family transposon (Table 9). The DNA extracted from field strains of *Enterococcus*, containing tetracycline resistance genes, was used as a positive control.

### 4.6. Statistical Analyses

The obtained results were analyzed with a Chi-square (X^2^) test to compare antimicrobial resistance and the distribution of resistance genes among the three *Enterococcus* species detected. The statistical significance threshold was set at a *p*-value ≤ 0.05.

## 5. Conclusions

The present study provides data on the enterococcal intestinal flora in healthy chickens never treated with antibiotics. In particular, the study focused on phenotypic and genotypic characters of antimicrobial resistance in *Enterococcus* strains. 

Resistances to most of the studied antimicrobials were found among the enterococci isolated from the investigated animals, and a relevant number of strains was multidrug resistant. The molecular analyses highlighted that chickens may harbor and be a source of tetracycline-resistant genes and transposons that facilitate the rapid transfer of the genes.

Having found enterococci with phenotypic and genotypic characters of antimicrobial resistance in young chickens bred in optimal conditions confirmed these bacteria as a relevant threat for poultry, which can develop pathologies responsible for economic losses. In addition, enterococci in chickens farmed for commercial purpose may contaminate eggs and meat that become a public health hazard. The hygiene of poultry farms is therefore fundamental to reduce the environment contamination with bacteria, the risk of infection by primary and secondary pathogens and consequently the need for antibiotic treatments.

## Figures and Tables

**Table 1:** Results obtained testing 98 *Enterococcus* spp. isolates versus 14 antimicrobials with the agar disk diffusion test.

Antimicrobials		Susceptible		Intermediate		Resistant	
Class	Molecules	N. Isolates	%	N. Isolates	%	N. Isolates	%
Ansamycin	RD	26	26.53	9	9.18	63	64.29
Phenicols	C	89	90.82	4	4.08	5	5.10
Oxazolidinones	LZD	87	88.78	6	6.12	5	5.10
Nitrofurantoins	F	93	94.90	1	1.02	4	4.08
Fluoroquinolones	CIP	39	39.80	41	41.84	18	18.37
	ENR	15	15.31	42	42.86	41	41.84
Glycopeptides	TEC	98	100	0	0.00	0	0.00
	VA	53	54.08	34	34.69	11	11.22
Macrolides	E	52	53.06	31	31.63	15	15.31
Streptogramins	QD	10	10.20	1	1.02	87	88.78
Penicillins	AMC	96	97.96	2	2.04	0	0.00
	AMP	91	92.86	0	0.00	7	7.14
Tetracyclines	TE	16	16.33	37	37.76	45	45.92
	TGC	68	69.39	0	0.00	30	30.61

Legend: RD, rifampicin; C, chloramphenicol; LZD, linezolid; F, nitrofurantoin; CIP, ciprofloxacin; ENR, enrofloxacin; TEC, teicoplanin; VA, vancomycin; E, erythromycin; QD, quinupristin–dalfopristin; AMC, amoxicillin–clavulanic acid; AMP, ampicillin; TE, tetracycline; TGC, tigecycline.

**Table 2:** Results of the agar disk diffusion test for 72 *Enterococcus faecium* strains.

Antimicrobials		Susceptible		Intermediate		Resistant	
Class	Molecules	N. Strains	%	N. Strains	%	N. Strains	%
Ansamycin	RD	13	18.06	2	2.78	57	79.17
Phenicols	C	69	95.83	1	1.39	2	2.78
Oxazolidinones	LZD	65	90.28	3	4.17	4	5.56
Nitrofurantoins	F	68	94.44	0	0.00	4	5.56
Fluoroquinolones	CIP	24	33.33	36	50.00	12	16.67
	ENR	7	9.72	32	44.44	33	45.83
Glycopeptides	TEC	72	100	0	0.00	0	0.00
	VA	36	50.00	26	36.11	10	13.89
Macrolides	E	45	62.50	22	30.56	5	6.94
Streptogramins	QD	3	4.17	0	0.00	69	95.83
Penicillins	AMC	70	97.22	2	2.78	0	0.00
	AMP	67	93.06	0	0.00	5	6.94
Tetracyclines	TE	6	8.33	31	43.06	35	48.61
	TGC	50	69.44	0	0.00	22	30.56

Legend. RD, rifampicin; C, chloramphenicol; LZD, linezolid; F, nitrofurantoin; CIP, ciprofloxacin; ENR, enrofloxacin; TEC, teicoplanin; VA, vancomycin; E, erythromycin; QD, quinupristin–dalfopristin; AMC, amoxicillin–clavulanic acid; AMP, ampicillin; TE, tetracycline; TGC, tigecycline.

**Table 3:** Results of the agar disk diffusion test for 23 *Enterococcus faecalis* strains.

Antimicrobials		Susceptible		Intermediate		Resistant	
Class	Molecules	N. Strains	%	N. Strains	%	N. Strains	%
Ansamycin	RD	13	56.52	7	30.43	3	13.04
Phenicols	C	18	78.26	2	8.70	3	13.04
Oxazolidinones	LZD	19	82.61	3	13.04	1	4.35
Nitrofurantoins	F	22	95.65	1	4.35	0	0.00
Fluoroquinolones	CIP	13	56.52	5	21.74	5	21.74
	ENR	7	30.43	9	39.13	7	30.43
Glycopeptides	TEC	23	100	0	0.00	0	0.00
	VA	15	65.22	7	30.43	1	4.35
Macrolides	E	4	17.39	9	39.13	10	43.48
Streptogramins	QD	5	21.74	0	0.00	18	78.26
Penicillins	AMC	23	100	0	0.00	0	0.00
	AMP	22	95.65	0	0.00	1	4.35
Tetracyclines	TE	9	39.13	5	21.74	9	39.13
	TGC	16	69.57	0	0.00	7	30.43

Legend: RD, rifampicin; C, chloramphenicol; LZD, linezolid; F, nitrofurantoin; CIP, ciprofloxacin; ENR, enrofloxacin; TEC, teicoplanin; VA, vancomycin; E, erythromycin; QD, quinupristin–dalfopristin; AMC, amoxicillin–clavulanic acid; AMP, ampicillin; TE, tetracycline; TGC, tigecycline.

**Table 4:** Results of the agar disk diffusion test for three *Enterococcus avium* strains.

Antimicrobials		Susceptible		Intermediate		Resistant	
Class	Molecules	N. Strains	%	N. Strains	%	N. Strains	%
Ansamycin	RD	0	0.00	0	0.00	3	100
Phenicols	C	2	66.66	1	33.33	0	0.00
Oxazolidinones	LZD	3	100	0	0.00	0	0.00
Nitrofurantoins	F	3	100	0	0.00	0	0.00
Fluoroquinolones	CIP	2	66.66	0	0.00	1	33.33
	ENR	1	33.33	1	33.33	1	33.33
Glycopeptides	TEC	3	100	0	0.00	0	0.00
	VA	2	66.66	1	33.33	0	0.00
Macrolides	E	3	100	0	0.00	0	0.00
Streptogramins	QD	2	66.66	1	33.33	0	0.00
Penicillins	AMC	3	100	0	0.00	0	0.00
	AMP	2	66.66	0	0.00	1	33.33
Tetracyclines	TE	1	33.33	1	33.33	1	33.33
	TGC	2	66.66	0	0.00	1	33.33

Legend: RD, rifampicin; C, chloramphenicol; LZD, linezolid; F, nitrofurantoin; CIP, ciprofloxacin; ENR, enrofloxacin; TEC, teicoplanin; VA, vancomycin; E, erythromycin; QD, quinupristin–dalfopristin; AMC, amoxicillin–clavulanic acid; AMP, ampicillin; TE, tetracycline; TGC, tigecycline.

**Table 5:** Identification of tetracycline resistance genes in relation to *Enterococcus* species.

Investigated Genes	*E. faecium* 72 Strains	*E. faecalis* 23 Strains	*E. avium* 3 Strains	Total 98 Strains
*tet*(M)	53 (73.61%)	9 (39.13%)	2 (66.67%)	64 (65.30%)
*tet*(L)	4 (5.56%)	6 (26.9%)	0 (0.00%)	10 (10.20%)
*tet*(O)	0 (0.00%)	2 (8.70%)	0 (0.00%)	2 (2.04%)
*tet*(K)	0 (0.00%)	0 (0.00%)	0 (0.00%)	0 (0.00%)
*Int-Tn*	8 (11.11%)	3 (13.04%)	1 (33.33%)	12 (12.24%)

**Table 6:** Phenotypic and genotypic resistance patterns for 72 *Enterococcus faecium* strains.

Number of Isolates	Resistance Patterns	Resistance Genes
2	RD	*tet*(M)
1	E-QD	*tet*(M)-*tet*(L)
1	E-QD	*tet*(M)-*Int-Tn*
2	ENR-QD	
2	QD-TE	*tet*(M)
1	RD-ENR	*tet*(M)
13	RD-QD	*tet*(M)
3	RD-QD	
2	RD-QD	*tet*(M)-*Int-Tn*
3	RD-ENR-QD	*tet*(M)
2	RD-QD-TE	
3	RD-QD-TE	*tet*(M)-*Int-Tn*
4	RD-QD-TE	*tet*(M)
2	RD-QD-TGC	
1	RD-QD-TGC	*tet*(M)
1	CIP-ENR-E-QD-HLSR-HLGR	
1	CIP-ENR-QD-TGC	
1	ENR-QD-TE-TGC	*tet*(M)
1	LZD-CIP-ENR-QD	
1	RD-CIP-QD-TE	*tet*(M)
2	RD-ENR-QD-TE	*tet*(M)
1	RD-ENR-QD-TE	*tet*(M)-*Int-Tn*
2	RD-ENR-QD-TE	
1	RD-ENR-QD-TGC	*tet*(M)
1	RD-QD-TE-TGC	
1	RD-QD-TE-TGC	*tet*(M)
1	C-F-CIP-ENR-QD	
1	CIP-ENR-QD-TE-TGC	*tet*(M)
1	F-ENR-QD-AMP-TE	*tet*(M)-*tet*(L)
2	RD-ENR-QD-TE-TGC	
3	RD-ENR-QD-TE-TGC	*tet*(M)
1	RD-LZD-ENR-QD-TGC	
1	F-ENR-E-QD-AMP-TE	*tet*(M)-*tet*(L)
1	F-ENR-QD-AMP-TE-TGC	*tet*(M)-*tet*(L)
3	RD-CIP-ENR-QD-TE-TGC	*tet*(M)
1	RD-CIP-ENR-QD-AMP-TE-TGC	*tet*(M)
1	RD-LZD-CIP-ENR-QD-AMP-TE-TGC	*tet*(M)-*Int-Tn*
1	RD-C-LZD-CIP-ENR-E-QD-TE-TGC	*tet*(M)

Legend: RD, rifampicin; C, chloramphenicol; LZD, linezolid; F, nitrofurantoin; CIP, ciprofloxacin; ENR, enrofloxacin; E, erythromycin; QD, quinupristin–dalfopristin; AMP, ampicillin; TE, tetracycline; TGC, tigecycline; HLSR, high-level streptomycin resistance; HLGR, high-level gentamicin resistance.

**Table 7:** Phenotypic and genotypic resistance patterns for 23 *Enterococcus faecalis* strains.

Number of Isolates	Resistance Patterns	Resistance Genes
4		
1	QD	*tet*(M)-*tet*(L)
1	QD	*tet*(M)
1	QD	
1	E-QD	
1	ENR-TGC	
1	QD-TE	*tet*(M)-*Int-Tn*
1	QD-TGC	
1	C-E-QD	*tet*(L)
1	C-E-QD	*tet*(M)-*tet*(L)
1	E-QD-TE-HLSR	*tet*(M)-*tet*(L)
1	E-QD-TE-HLSR	*tet*(L)
1	E-QD-TE-HLSR	
1	E-QD-TGC-HLSR	*tet*(M)-*tet*(L)
1	CIP-ENR-E-QD	
1	RD-ENR-QD-TE-TGC	*tet*(M)-*Int-Tn*
1	CIP-ENR-E-QD-TE-TGC-HLGR	*tet*(O)
1	RD-CIP-ENR-QD-TE-TGC	*tet*(M)-*Int-Tn*
1	C-LZD-CIP-ENR-E-QD-TE-HLGR	*tet*(O)
1	RD-CIP-ENR-QD-AMP-TE-TGC	*tet*(M)

Legend: RD, rifampicin; C, chloramphenicol; LZD, linezolid; CIP, ciprofloxacin; ENR, enrofloxacin; E, erythromycin; QD, quinupristin–dalfopristin; AMP, ampicillin; TE, tetracycline; TGC, tigecycline; HLSR, high-level streptomycin resistance; HLGR, high-level gentamicin resistance.

**Table 8:** Phenotypic and genotypic resistance patterns for three *Enterococcus avium* strains.

Number of Isolates	Resistance Patterns	Resistance Genes
1	RD	
1	RD	*tet*(M)
1	RD-CIP-ENR-AMP-TE-TGC	*tet*(M)-*Int-Tn*

Legend. RD, rifampicin; CIP, ciprofloxacin; ENR, enrofloxacin; AMP, ampicillin; TE, tetracycline; TGC, tigecycline.

**Table 9:** Primers and protocols employed in the PCR analyses to detect genes encoding for resistance to tetracyclines.

Target Gene	Sequence (5′-3′)	Annealing T (°C)	Amplicon Size (bp)	Reference
*tet*(M)	F: GTGGACAAAGGTACAACGAG R: CGGTAAAGTTCGTCACAC	61	406	[60]
*tet*(L)	F: TGGTGGAATGATAGCCCATT R: CAGGAATGACAGCACGCTAA	61	229	[60]
*tet*(O)	F: AACTTAGGCATTCTGGCTCAC R: TCCCACTGTTCCATATCGTCA	61	515	[60]
*tet*(K)	F: GATCAATTGTAGCTTTAGGTGAAGG R: TTTTGTTGATTTACCAGGTACCATT	61	155	[60]
*Int-Tn*	F: GCGTGATTGTATCTCACT R: GACGCTCCTGTTGCTTCT	50	1028	[52]

## Data Availability

Data are reported in the manuscript.

## References

[1] Cheng W.N., Han S.G. (2020). Bovine mastitis: Risk factors, therapeutic strategies, and alternative treatments—A review. Asian Australas. J. Anim. Sci..

[2] Wood M.W., Lepold A., Tesfamichael D., Lasarev M.R. (2020). Risk factors for enterococcal bacteriuria in dogs: A retrospective study. J. Vet. Intern. Med..

[3] Robbins K.M., Suyemoto M.M., Lyman R.L., Martin M.P., Barnes H.J., Borst L.B. (2012). An outbreak and source investigation of enterococcal spondylitis in broilers caused by *Enterococcus cecorum*. Avian Dis..

[4] Seputiene V., Bogdaite A., Ruzauskas M., Suziedeliene E. (2012). Antibiotic resistance genes and virulence factors in *Enterococcus faecium* and *Enterococcus faecalis* from diseased farm animals: Pigs, cattle and poultry. Pol. J. Vet. Sci..

[5] Hollenbeck B.L., Rice L.B. (2012). Intrinsic and acquired resistance mechanisms in enterococcus. Virulence.

[6] Kristich C.J., Rice L.B., Arias C.A., Gilmore M.S., Clewell D.B., Ike Y., Shankar N. (2014). Enterococcal Infection–Treatment and Antibiotic Resistance. Enterococci: From Commensals to Leading Causes of Drug Resistant Infection.

[7] García-Solache M., Rice L.B. (2019). The *Enterococcus*: A Model of Adaptability to Its Environment. Clin. Microbiol. Rev..

[8] Hegstad K., Mikalsen T., Coque T.M., Werner G., Sundsfjord A. (2010). Mobile genetics elements and their contribution to the emergence of antimicrobial resistant *Enterococcus faecalis* and *Enterococcus faecium*. Clin. Microbiol. Infect..

[9] Alzahrani O.M., Fayez M., Alswat A.S., Alkafafy M., Mahmoud S.F., Al-Marri T., Almuslem A., Ashfaq H., Yusuf S. (2022). Antimicrobial Resistance, Biofilm Formation, and Virulence Genes in *Enterococcus* Species from Small Backyard Chicken Flocks. Antibiotics.

[10] Staley C., Dunny G.M., Sadowsky M.J. (2014). Environmental and animal-associated enterococci. Adv. Appl. Microbiol..

[11] Cagnoli G., Bertelloni F., Interrante P., Ceccherelli R., Marzoni M., Ebani V.V. (2022). Antimicrobial-Resistant *Enterococcus* spp. in Wild Avifauna from Central Italy. Antibiotics.

[12] Kilonzo-Nthenge A., Brown A., Nahashon S.N., Long D. (2015). Occurrence and antimicrobial resistance of enterococci isolated from organic and conventional retail chicken. J. Food Prot..

[13] Ayeni F.A., Odumosu B.T., Oluseyi A.E., Ruppitsch W. (2016). Identification and prevalence of tetracycline resistance in enterococci isolated from poultry in Ilishan, Ogun State, Nigeria. J. Pharm. Bioallied Sci..

[14] de Jong A., Simjee S., El Garch F., Moyaert H., Rose M., Youala M., Dry M. (2018). Antimicrobial susceptibility of enterococci recovered from healthy cattle, pigs and chickens in nine EU countries (EASSA Study) to critically important antibiotics. Vet. Microbiol..

[15] Makarov D.A., Ivanova O.E., Pomazkova A.V., Egoreva M.A., Prasolova O.V., Lenev S.V., Gergel M.A., Bukova N.K., Karabanov S.Y. (2022). Antimicrobial resistance of commensal *Enterococcus faecalis* and *Enterococcus faecium* from food-producing animals in Russia. Vet. World.

[16] Souillard R., Laurentie J., Kempf I., Le Caër V., Le Bouquin S., Serror P., Allain V. (2022). Increasing incidence of *Enterococcus*-associated diseases in poultry in France over the past 15 years. Vet. Microbiol..

[17] Nielsen S.S., Bicout D.J., Calistri P., Canali E., Drewe J.A., Garin-Bastuji B., Gonzales Rojas J.L., Gortázar C., Herskin M., EFSA AHAW Panel (EFSA Panel on Animal Health and Welfare) (2022). Assessment of listing and categorisation of animal diseases within the framework of the Animal Health Law (Regulation (EU) No 2016/429): Antimicrobial-resistant *Enterococcus faecalis* in poultry. EFSA J..

[18] Bertelloni F., Salvadori C., Moni A., Cerri D., Mani P., Ebani V.V. (2015). Antimicrobial resistance in *Enterococcus* spp. isolated from laying hens of backyard poultry floks. Ann. Agric. Environ. Med..

[19] Hayes J.R., McIntosh A.C., Qaiyumi S., Johnson J.A., English L.L., Carr L.E., Wagner D.D., Joseph S.W. (2001). High-frequency recovery of quinupristin-dalfopristin-resistant *Enterococcus faecium* isolates from the poultry production environment. J. Clin. Microbiol..

[20] Tamai S., Suzuki Y. (2023). Diversity of Fecal Indicator Enterococci among Different Hosts: Importance to Water Contamination Source Tracking. Microorganisms.

[21] Aarestrup F.M., Agerso Y., Gerner-Smidt P., Madsen M., Jensen L.B. (2000). Comparison of antimicrobial resistance phenotypes and resistance genes in *Enterococcus faecalis* and *Enterococcus faecium* from humans in the community, broilers, and pigs in Denmark. Diagn. Microbiol. Infect. Dis..

[22] Maasjost J., Mühldorfer K., Cortez de Jäckel S., Hafez H.M. (2015). Antimicrobial Susceptibility Patterns of *Enterococcus faecalis* and *Enterococcus faecium* Isolated from Poultry Flocks in Germany. Avian Dis..

[23] Stępień-Pyśniak D., Marek A., Banach T., Adaszek Ł., Pyzik E., Wilczyński J., Winiarczyk S. (2016). Prevalence and antibiotic resistance of *Enterococcus* strains isolated from poultry. Acta Vet. Hung..

[24] Semedo-Lemsaddek T., Bettencourt Cota J., Ribeiro T., Pimentel A., Tavares L., Bernando F., Oliveira M. (2021). Resistance and virulence distribution in enterococci isolated from broilers reared in two farming systems. Ir. Vet. J..

[25] Fertner M.E., Olsen R.H., Bisgaard M., Christensen H. (2011). Transmission and genetic diversity of *Enterococcus faecalis* among layer chickens during hatch. Acta Vet..

[26] Chai W.L., Hamimah H., Cheng S.C., Sallam A.A., Abdullah M. (2007). Susceptibility of *Enterococcus faecalis* biofilm to antibiotics and calcium hydroxide. J. Oral Sci..

[27] Diarra M.S., Rempel H., Champagne J., Masson L., Pritchard J., Topp E. (2010). Distribution of antimicrobial resistance and virulence genes in *Enterococcus* spp. and characterization of isolates from broiler chickens. Appl. Environ. Microbiol..

[28] Song H., Bae Y., Jeon E., Kwon Y., Joh S. (2019). Multiplex PCR analysis of virulence genes and their influence on antibiotic resistance in *Enterococcus* spp. isolated from broiler chicken. J. Vet. Sci..

[29] Solway S., Vincent L., Tian N., Woodford N., Bendall R. (2003). Isolation of streptogramin-resistant Enterococcus faecium from human and non-human sources in a rural community. J. Antimicrob. Chemother..

[30] Holmberg A., Rasmussen M. (2014). Antibiotic regimens with rifampicin for treatment of Enterococcus faecium in biofilms. Int. J. Antimicrob. Agents.

[31] Chen W., Wang Q., Wu H., Xia P., Tian R., Li R., Xia L. (2024). Molecular epidemiology, phenotypic and genomic characterization of antibiotic-resistant enterococcal isolates from diverse farm animals in Xinjiang, China. Sci. Total Environ..

[32] Cavaco L.M., Bernal J.F., Zankari E., Léon M., Hendriksen R.S., Perez-Gutierrez E., Aarestrup F.M., Donado-Godoy P. (2017). Detection of linezolid resistance due to the optrA gene in *Enterococcus faecalis* from poultry meat from the American continent (Colombia). J. Antimicrob. Chemother..

[33] Yoon S., Kim Y.B., Seo K.W., Ha J.S., Noh E.B., Lee Y.J. (2020). Characteristics of linezolid-resistant *Enterococcus faecalis* isolates from broiler breeder farms. Poult. Sci..

[34] World Health Organization (WHO) 19th WHO Model List of Essential Medicines (April 2015). https://www.iccp-portal.org/system/files/resources/EML2015_8-May-15.pdf.

[35] Noh E.B., Kim Y.B., Seo K.W., Son S.H., Ha J.S., Lee Y.J. (2020). Antimicrobial resistance monitoring of commensal *Enterococcus faecalis* in broiler breeders. Poult. Sci..

[36] Bae S.H., Yoon S., Kim K., Kim Y.B., Lee Y.J. (2021). Comparative Analysis of Chloramphenicol-Resistant *Enterococcus faecalis* Isolated from Dairy Companies in Korea. Vet. Sci..

[37] Hummel A., Holzapfel W.H., Franz C.M. (2007). Characterisation and transfer of antibiotic resistance genes from enterococci isolated from food. Syst. Appl. Microbiol..

[38] Tatsing Foka F.E., Ateba C.N. (2019). Detection of Virulence Genes in Multidrug Resistant Enterococci Isolated from Feedlots Dairy and Beef Cattle: Implications for Human Health and Food Safety. Biomed. Res. Int..

[39] CDC (2019). Antibiotic Resistance Threats in The United States. https://www.cdc.gov/drugresistance/pdf/threats-report/2019-ar-threats-report-508.pdf.

[40] ECDC, WHO Europe (2022). Antimicrobial Resistance Surveillance in Europe 2022 (2020 Data). https://www.ecdc.europa.eu/sites/default/files/documents/ECDC-WHO-AMR-report.pdf.

[41] Liu C., Yoon E.J., Kim D., Shin J.H., Shin J.H., Shin K.S., Kim Y.A., Uh Y., Kim H.S., Kim Y.R. (2019). Antimicrobial resistance in South Korea: A report from the Korean global antimicrobial resistance surveillance system (Kor-GLASS) for 2017. J. Infect. Chemother..

[42] Japan Nosocomial Infections Surveillance (JANIS) Annual Open Report 2020 (All Facilities). CLSI 2012 Version. Clinical Laboratory Division 2021. https://janis.mhlw.go.jp/english/report/open_report/2020/3/1/ken_Open_Report_Eng_202000_clsi2012.pdf.

[43] Yamaguchi R., Yamamoto T., Okamoto K., Harada S., Echizenya M., Tsutsumi T., Takada T. (2023). Teicoplanin and vancomycin as treatment for glycopeptide-susceptible Enterococcus faecium bacteraemia: A propensity score-adjusted non-inferior comparative study, J. Antimicrob. Chemother..

[44] Lee Y.J., Kim K., Lee Y.J. (2023). Dissemination and characteristics of high-level erythromycin-resistant *Enterococcus faecalis* from bulk tank milk of dairy companies in Korea. Can. J. Vet. Res..

[45] Sanlibaba P., Senturk E. (2018). Prevalence, characterization, and antibiotic resistance of enterococci from traditional cheeses in turkey. Int. J. Food Prop..

[46] Torres C., Alonso C.A., Ruiz-Ripa L., León-Sampedro R., Del Campo R., Coque T.M. (2018). Antimicrobial Resistance in *Enterococcus* spp. of animal origin. Microbiol. Spectr..

[47] Poeta P., Costa D., Rodrigues J., Torres C. (2006). Antimicrobial resistance and the mechanisms implicated in faecal enterococci from healthy humans, poultry and pets in Portugal. Int. J. Antimicrob. Agents.

[48] Ribeiro J., Silva V., Monteiro A., Vieira-Pinto M., Igrejas G., Reis F.S., Barros L., Poeta P. (2023). Antibiotic Resistance among Gastrointestinal Bacteria in Broilers: A Review Focused on *Enterococcus* spp. and *Escherichia coli*. Animals.

[49] Furtula V., Jackson C.R., Farrell E.G., Barrett J.B., Hiott L.M., Chambers P.A. (2013). Antimicrobial resistance in *Enterococcus* spp. isolated from environmental samples in an area of intensive poultry production. Int. J. Environ. Res. Public Health.

[50] Manoil D., Cerit E.E., Fang H., Durual S., Brundin M., Belibasakis G.N. (2024). Profiling Antibiotic Susceptibility among Distinct Enterococcus faecalis Isolates from Dental Root Canals. Antibiotics.

[51] Giovanetti E., Brenciani A., Lupidi R., Roberts M.C., Varaldo P.E. (2003). Presence of the *tet*(O) gene in erythromycin- and tetracycline-resistant strains of *Streptococcus pyogenes* and linkage with either the *mef*(A) or the *erm*(A) gene. Antimicrob. Agents Chemother..

[52] Doherty N., Trzcinski K., Pickerill P., Zawadzki P., Dowson C.G. (2000). Genetic diversity of the *tet*(M) gene in tetracycline-resistant clonal lineages of *Streptococcus pneumoniae*. Antimicrob. Agents Chemother..

[53] Wiśniewski P., Zakrzewski A., Chajęcka-Wierzchowska W., Zadernowska A. (2024). Possibility of transfer and activation of ‘silent’ tetracycline resistance genes among *Enterococcus faecalis* under high-pressure processing. Food Microbiol..

[54] Ding J., Dai R., Yang L., He C., Xu K., Liu S., Zhao W., Xiao L., Luo L., Zhang Y. (2017). Inheritance and Establishment of Gut Microbiota in Chickens. Front. Microbiol..

[55] (2018). Performance Standards for Antimicrobial Disk Susceptibility Tests, 13th ed..

[56] (2023). Performance Standards for Antimicrobial Susceptibility Testing, 33rd ed..

[57] EUCAST (The European Committee on Antimicrobial Susceptibility Testing) (2023). Breakpoint Tables for Interpretation of MICs and Zone Diameters, Version 13.0.

[58] (2018). Methods for Dilution Antimicrobial Susceptibility Tests for Bacteria That Grow Aerobically, 11th ed..

[59] Magiorakos A.P., Srinivasan A., Carey R.B., Carmeli Y., Falagas M.E., Giske C.G., Harbarth S., Hindler J.F., Kahlmeter G., Olsson-Liljequist B. (2012). Multidrug-resistant, extensively drug-resistant and pandrug-resistant bacteria: An international expert proposal for interim standard definitions for acquired resistance. Clin. Microbiol. Infect..

[60] Malhotra-Kumar S., Lammens C., Piessens J., Goossens H. (2005). Multiplex PCR for simultaneous detection of macrolide and tetracycline resistance determinants in streptococci. Antimicrob. Agents Chemother..

